# The effects of right temporoparietal junction stimulation on embodiment, presence, and performance in teleoperation

**DOI:** 10.3934/Neuroscience.2024022

**Published:** 2024-09-10

**Authors:** Valentina Cesari, Graziella Orrù, Andrea Piarulli, Alessandra Vallefuoco, Franca Melfi, Angelo Gemignani, Danilo Menicucci

**Affiliations:** 1 Department of Surgical, Medical and Molecular Pathology and Critical Care Medicine, University of Pisa, Pisa, Italy; 2 Clinical Psychology Branch, Azienda Ospedaliero-Universitaria Pisana, Pisa, Italy

**Keywords:** brain modulation, consciousness, ownership, performance, presence, tDCS, virtual reality

## Abstract

Embodiment (the sensation that arises when the properties of an external instrument are processed as if they are the attributes of one's own biological body) and (tele)presence (the sensation of being fully engaged and immersed in a location other than the physical space occupied by one's body) sustain the perception of the physical self and potentially improve performance in teleoperations (a system that enables human intelligence to control robots and requires implementing an effective human-machine interface). Embodiment and presence may be interdependent and influenced by right temporo-parietal junction (rTPJ) activity. We investigated the interplay between embodiment, (tele)presence, and performance in teleoperation, focusing on the role of the rTPJ. Participants underwent a virtual reality task with transcranial direct current stimulation (tDCS) twice, receiving either active or sham stimulation. Behavioral measures (driving inaccuracy, elapsed time in the lap, time spent in attentional lapses, short-term self-similarity, and long-term self-similarity), perceived workload (mental demand, physical demand, temporal demand, own performance, effort, and frustration), embodiment's components (ownership, agency, tactile sensations, location, and external appearance), and presence's components (realism, possibility to act, quality of interface, possibility to examine, self-evaluation of performance, haptic, and sounds) were assessed. The results showed that rTPJ stimulation decreased perceived ownership but enhanced presence with changes in the complexity of visuomotor adjustments (long and short-term self-similarity indices). Structural equation modeling revealed that embodiment increased visuomotor inaccuracy (a composite variable of overall performance, including deviations from the optimal trajectory and the time taken to complete the task), presence reduced workload, and workload increased inaccuracy. These results suggested a dissociation between embodiment and presence, with embodiment hindering performance. Prioritizing virtual integration may lower human performance, while reduced workload from presence could aid engagement. These findings emphasize the intricate interplay between rTPJ, subjective experiences, and performance in teleoperation.

## 1. Introduction

The ability to control actions in remote environments is a crucial aspect of teleoperations, which enables human control of tools, even robots, through a human-machine interface, involving a master arm guided by the operator and a slave arm that replicates the master's movements [1],[2]. This system is conceived to perform complex tasks in challenging and unstructured environments, including extreme conditions such as space, nuclear facilities, and underwater environments. Teleoperation is crucial for applications such as search and rescue, disaster relief, explosive ordnance disposal, and other complex, unpredictable, or hazardous environments, including minimally invasive surgery [3]–[5]. When performing such intricate tasks using telemanipulators, e.g., robotic arms, individuals must experience a sense of physical presence at the interaction point. This sensation helps to make the actions feel as natural and intuitive as if they were being performed directly [6]. This is the sense of embodiment, defined as the sensation that arises when the properties of an external instrument are processed as if they are the attributes of one's own biological body [7] and it can potentially mitigate the challenge posed by a mismatch between the human body and teleoperated effectors. Research indicates that this sense of embodiment can diminish the individual's susceptibility to inconsistencies in size, movement, degrees of freedom, and other factors associated with teleoperated devices [6]. The sense of embodiment encompasses three major components: the sense of self-location, involving the perception of being located in a remote space; the sense of ownership, entailing the belief that non-bodily objects, such as tools, are integral parts of one's own body; the sense of agency, encompassing the feeling of being the author of an observed action of motor control [6],[7].

Indeed, the sense of embodiment has been shown to enhance the sense of presence [8],[9]. Presence, in this context, refers to the sensation of being fully engaged and immersed in a location other than the physical space occupied by one's body, commonly known as telepresence [10]. Telepresence, or the sense of telepresence, is understood as the subjective feeling of being in a virtual environment, temporarily unaware of one's actual surroundings, location, and the technology facilitating the flow of virtual sensory input [11].

Both the senses of embodiment and presence have been identified as being sustained by neuroanatomical hubs involved in multisensory integration. These hubs may be crucial in determining the qualitative aspect of conscious contents and facilitating a non-ordinary state of consciousness. Within these hubs, the right temporoparietal junction (rTPJ) plays a significant role in constructing a sense of a bodily self by integrating information from various sensory channels, including vision, proprioception, hearing, touch, and motor control [12],[13]. The TPJ area is implicated in various facets of embodiment, such as the sense of agency, self-other distinction, body ownership, and body imagery [14]. Notably, experiences of disembodiment have been reported when the activity in this area or its connections are altered [13],[15],[16]. Significant research has shown that non-invasive brain stimulation targeting the right temporoparietal junction can modulate the sense of embodiment by influencing multisensory integration. Non-invasive brain stimulation is a promising tool for promoting neuroplasticity by altering brain activity in specific cortical regions and related brain networks [17],[18]. These techniques offer effective neuromodulation at a reasonable cost [19]. The most used methods of non-invasive brain stimulation are transcranial magnetic stimulation, which uses magnetic fields to stimulate neural activity, and transcranial electrical stimulation, including transcranial direct current stimulation (tDCS), which applies a low-intensity electrical current to modulate brain activity and function. Notably, transcranial magnetic stimulation targeting the rTPJ has been found to alter the perceptual boundaries of the body, inducing a pronounced illusionary experience [13],[16]. Moreover, tDCS applied to the rTPJ has been shown to enhance the sense of agency over avatars in virtual reality (VR) settings [20], and to enhance proprioceptive displacement in the rubber hand illusion paradigm, with the effect modulated by the temporal congruency of the visuo-tactile stimulation [21].

Further showing the potential of non-invasive brain stimulation to modulate multisensory integration and embodiment, Lapenta et al. [22] found that tDCS over the superior temporal cortex modulates performance in multisensory tasks with gender-specific effects, Marques et al. [23] showed that tDCS can modulate auditory-visual integration in the McGurk illusion, and Lira et al. [24] revealed that tDCS accelerates body ownership in the rubber hand illusion by enhancing parietal cortex function.

Due to the crucial role of embodiment in the bodily self-component of consciousness, Toet and colleagues [6] have proposed a groundbreaking hypothesis suggesting its contribution to improve dexterity in teleoperation. However, studies investigating embodiment have predominantly focused on body illusions, such as the Rubber Hand Illusion [6], and its role in individuals' performance remains insufficiently explored.

Building on Toet's hypothesis, the present study aims to investigate how the sense of embodiment influences individuals' performance in teleoperation tasks. To achieve this goal, we applied tDCS to participants while they engaged in a VR driving task. We hypothesize that a stronger sense of embodiment will improve teleoperation performance. In addition, we expect that modulating the sense of embodiment and presence through tDCS targeting rTPJ will enhance these effects, thereby clarifying the relationship between embodiment and teleoperation effectiveness.

## 2. Materials and methods

### 2.1. Participants

We received ethical approval from the Bio-Ethical Committee of the University of Pisa on 30/01/2023 (protocol number: 0012001/2023). The study was conducted in accordance with the Declaration of Helsinki.

Twenty-six healthy young volunteers (13 cisgender females and 13 cisgender males; mean age ± SD: 24.5 ± 2) were enrolled on a voluntary basis.

The sample size was determined using a priori power analysis conducted with G*Power (ver. 3.1.9.7; Heinrich-Heine-Universität Düsseldorf, Düsseldorf, Germany; http://www.gpower.hhu.de/). The type of tDCS session was used as the within-subject factor for this analysis, with an alpha level of .05, power set at .80, and an effect size of f = 0.25. This effect size was selected based on similar studies that reported comparable effects in the context of non-invasive brain stimulation (see [25]–[27], for examples). These parameters were used in the Repeated Measures Analysis of Variance (ANOVA), as described in section 2.6, to calculate the required sample size.

### 2.2. Experimental overview

**Figure 1.**
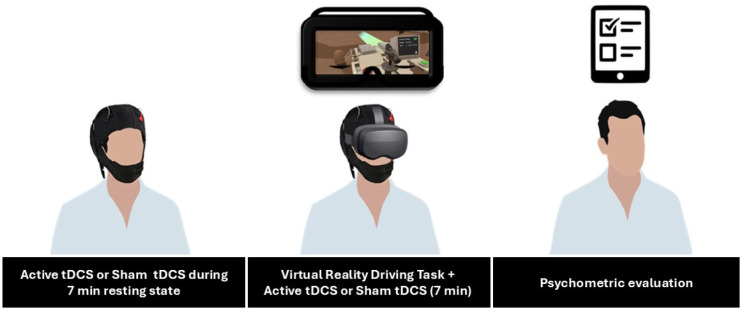
Overview of the experimental protocol.

The experimental protocol (Figure 1) consisted of two sessions, spaced one week apart, during which volunteers performed a VR driving simulation task. In one session, they received tDCS targeting the rTPJ (active session), while in the other session, a placebo (sham) stimulation was administered. The stimulation began 7 minutes before the VR task and continues throughout its duration (7 minutes). This approach was supported by research indicating that tDCS can modulate motor learning in a polarity- and time-dependent manner. Following Stagg et al. [28] anodal tDCS applied during a motor task enhances learning speed, while cathodal tDCS impedes it. Conversely, when tDCS is applied before the task, both anodal and cathodal stimulation results in slower learning. Therefore, we chose to apply tDCS simultaneously with the VR task to leverage these time-dependent effects.

The sequence of the sessions was randomized, and the experiment was conducted in a double-blind manner, ensuring that neither the operator nor the volunteer was aware of whether the session involved active or sham stimulation.

Following the task completion, participants were instructed to complete a self-report test battery assessing their level of embodiment (Avatar Embodiment), their sense of telepresence (Presence Questionnaire), and their perceived workload (NASA TLX) during the task.

### 2.3. Transcranial Direct Current Stimulation

During the active session, the rTPJ was stimulated via tDCS following the methodology outlined by Skola and Liarokapis [20]: the anodal current was applied using an electrode placed at the CP6 location, while the return electrode was positioned at the vertex (CZ position). For the placement of the electrodes, we used anatomical landmarks in accordance with the international 10–20 system for EEG recording.

**Figure 2.**
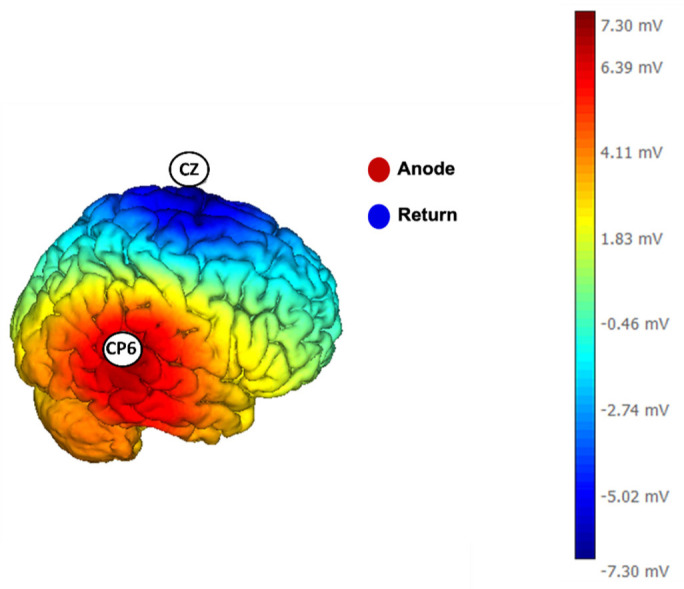
3D representation of the estimated electric field based on the cerebral hemispheres modeling (source: Neuroelectrics NIC software).

The anodal current was delivered for a total of 14 minutes (7 minutes during the resting state and 7 minutes during the task). A model illustrating the distribution of electrical potential over the cortical mantle, based on the stimulation configuration provided by the proprietary software of the stimulator (NIC software, Neuroelectrics, Spain), is reported in Figure 2.

Stimulation was performed using 2 AgCl gel-based electrodes (NG Pistim electrode, Neuroelectrics, Barcelona, Spain) with a radius of 1 cm. The stimulation current was set to 1 mA, and the duration of stimulation was 14 minutes, which included 5-seconds of ramp-up and 5-seconds of ramp-down. The duration of stimulation was set based on literature indicating that it is expected to modulate the targeted brain area for at least an hour post-stimulation [29]. The intensity of stimulation was based on recent findings indicating that low intensities can be effective. Specifically, Khalil et al. [30] showed that 1 mA tDCS provided robust effects without additional benefits from increasing the intensity to 1.5 mA. Additionally, Santiesteban et al. [31],[32] showed that 1 mA stimulation targeting the temporoparietal junction is effective while maintaining participant comfort and safety, aligning with best practices for minimizing adverse effects. Electrode impedance was maintained below 10 KOhm to ensure participant comfort.

The sham stimulation procedure mirrored the active stimulation procedure from both the operator's and the subject's perspective. This included attaching the electrode cap with two gel electrodes and performing an impedance check. Electrode positioning was consistent for both active and sham tDCS conditions. However, after passing the impedance check, the stimulator began a process that closely resembled real stimulation, but with one significant difference: in the sham condition, the tDCS device was turned off after the initial ramp-up period, simulating the sensation of active stimulation but without delivering the actual electrical current. In the sham session, the tDCS was ON only during the 5-second ramp-up and 5-second ramp-down phases, with no stimulation delivered during the middle period.

The Starstim 8 device (Neuroelectrics, Spain) was employed for the stimulation procedure. This was carried out using its proprietary software (NIC).

An investigator (DM), distinct from the one operating on the volunteer, was responsible for configuring the stimulator in either active or sham mode for each session. Additionally, this investigator managed the database containing the records of session details and the associated stimulation modes.

### 2.4. Virtual Reality Task

#### 2.4.1. Apparatus

Volunteers were engaged in the VR driving simulation [33], by wearing a VR headset and holding one controller from the HTC Vive system (www.vive.com/). Participants were seated in a quiet environment with no auditory or visual distractions. In the virtual scenario, participants navigated a barren, alien-like terrain using a rover that resembled a lunar vehicle. A single HTC Vive controller, assigned based on the participant's dominant hand, was employed to control the rover. Participants had to interact with an onboard joystick using the right or left controller by moving it to bring the virtual hand closer to the joystick and pressing the trigger button to grasp it. Once the joystick was held, participants could move it to control the rover's speed and direction, enabling precise control over the vehicle's movement. This virtual scenario was designed to minimize distractions, introduce task monotony, standardize driving skills across participants using an unfamiliar control lever, and motivate volunteer participation. Haptic feedback was provided when volunteers encountered rocks along the rover's trajectory to enhance the realism of the virtual experience. Otherwise, no additional sensory feedback was provided.

Participants were instructed to follow a colored guide (green) on the ground indicating the curvilinear path they were to follow with the rover and were given instructions on how to use controllers to manipulate the rover's direction and speed.

The goal of the task was to follow the green guideline indicating the path, which appeared gradually to indicate directional changes and required constant attention to adjust as accurately as possible while moving forward as quickly as possible. The task was structured as a single trial lasting approximately 7 minutes. The VR software recorded rover wheel positions over time at a sampling rate of 27 Hz, synchronized with expected trajectories, providing data on driving performance.

**Figure 3.**
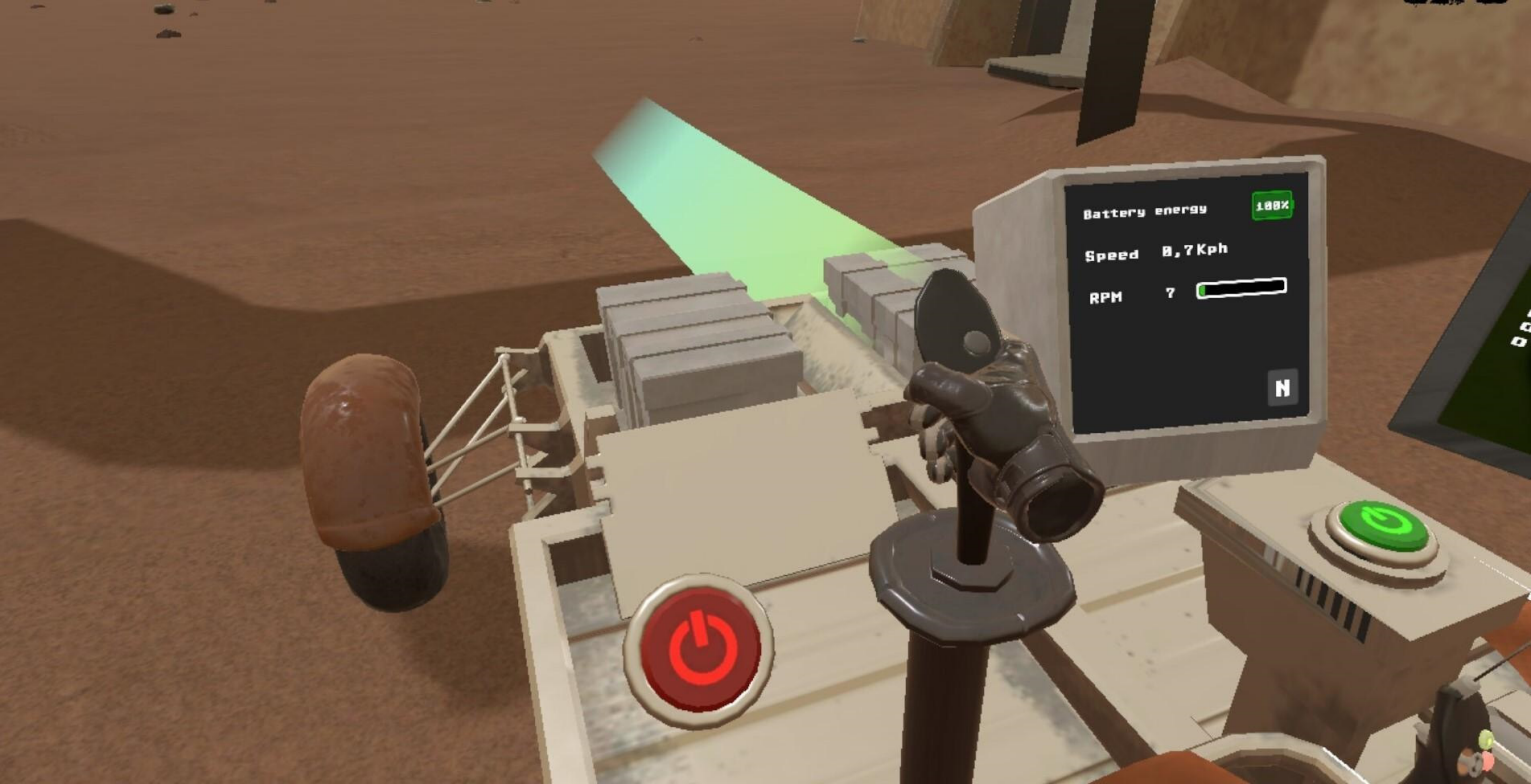
Virtual reality scenario during the visuomotor driving task. For a detailed description, refer to [33].

#### 2.4.2. Behavioral measures of performance

Visuomotor coordination involves fine adjustments of movements to reach a target. In the VR driving task, the differences between the expected and actual trajectories (departures) indicate the quality of visuomotor coordination [34]. The departure can be estimated for each sampling time point during the task, thus obtaining the time series of departures.

Visuomotor coordination during the driving task was assessed using measures introduced by Bufano et al. [33]. These measures were derived from the time series of departures calculated over an entire lap (Figure 3) and included: 1) Driving inaccuracy; 2) elapsed time in the lap; 3) time spent in attentional lapses; 4) short-term self-similarity; and 5) long-term self-similarity in the departure time series. For the self-similarity indices, they were derived from the Detrended Fluctuation Analysis [35],[36]. The short-term self-similarity relates to the 0.2–2s time scale, whereas the long-term one relates to the 2–20s time scale. The two distinct indices were introduced to take into account the characteristic knee exhibited by the relation between the amplitude of fluctuations and time scales [37].

### 2.5. Psychometric tests

#### 2.5.1. Avatar Embodiment

The Avatar Embodiment [38] is a self-report questionnaire consisting of 25 items on a 7-point Likert scale ranging from a score of “strongly disagree” (−3), “disagree” (−2), “somewhat disagree” (−1), “neither agree nor disagree” (0), “somewhat agree” (1), “agree” (2) to “strongly agree” (3).

The factors examined in the questionnaire were the following:

- Body Ownership: perception of ownership over a virtual or augmented body;
- Agency and Motor Control: participant's ability to control parts or the entirety of a virtual body through movement tracking;
- Tactile Sensations: sensory feedback augmenting the feeling of embodiment, often through tactile stimulation;
- Location: alignment of one's perceived body location with that of a virtual body, important for the illusion of embodiment;
- External Appearance: degree of resemblance between the subject's avatar and themselves;
- Response to External Stimuli: participant's reaction to events threatening their body or the avatar's body, resembling real body responses, akin to the rubber hand illusion.

In our experiment, we adapted the questionnaire by removing items associated with the “response to external stimuli” scale, as they were unsuitable for the task. Additionally, we employed the “back-translation” method to translate the scale into Italian while preserving the original psychometric properties [39].

#### 2.5.2. Presence questionnaire

The Presence questionnaire [40] was adapted from the original version developed by Witmer & Singer [41]. After undergoing back-translation verification, it was submitted in Italian. This tool aims to measure the level of immersion of a subject in a particular virtual environment. The questionnaire includes 24 items rated on a 7-point Likert scale, assessing the extent of agreement exhibited by subjects for each item. The questionnaire encompasses the following factors:

- Realism: the similarity between virtual and real environments;
- Possibility to act: the ability to explore and manipulate information in VR;
- Quality of interface: the degree to which the individual experiences delay between avatar and actual movement;
- Possibility to examine: capability of approaching and inspecting virtual objects from various angles;
- Self-evaluation of performance: the self-awareness experienced in performing tasks in VR;
- Sounds: the perception of sounds in VR;
- Haptic: indicates the perception of haptic feedback in VR.

#### 2.5.3. NASA TLX

The NASA TLX [42] is a self-assessment questionnaire providing an overall perceived workload score, based on a weighted average of six subscales:

- Mental Demand;
- Physical Demand;
- Temporal Demand;
- Own Performance;
- Effort;
- Frustration.

Participants rate the perceived workload experienced during prior completion of cognitive tasks by selecting a score ranging from 0 to 100 for each of the six subscales; higher scores indicate a greater perceived workload. In this study, the perceived workload rating was assessed using the PEBL software version of NASA TLX (PEBL TLX; [43]).

#### 2.5.4. Assessment of Sensations During tDCS session

To assess the sensations experienced during tDCS stimulation, we employed a brief follow-up interview based on an Adverse Effects Questionnaire adapted from Poreisz et al. [44]. This questionnaire was conceived to monitor common adverse effects associated with tDCS, including tingling, itchiness, and fatigue. Participants were interviewed 1 hour after each tDCS session (after completion of questionnaires) to report any experience sensations and their duration.

### 2.6. Statistical Analyses

As a summary, at the end of each experiment, we obtained:

1) Five behavioral measures of performance (2.4.2).

2) Twelve psychometric subscales from the Avatar Embodiment and the Presence questionnaires (2.5.1 and 2.5.2).

3) Six subscales from the NASA TLX scales (2.5.3).

To examine the impact of rTPJ stimulation, the scores of each measure underwent a Repeated Measure ANOVA (2.6.1).

For identifying the relationship between constructs, we estimated causal and simultaneous relationships among psychometric and behavioral indices of performance via Partial Least Squares Structural Equation Modeling (PLS-SEM) with embodiment set as the exogenous variable (2.6.3) according to its central role in dexterity in teleoperation [6].

In the face of the high number of indices, we applied a data reduction procedure (Exploratory Factor analysis, EFA, and Confirmatory Factor analysis, CFA, see section 2.6.2) that resulted in two components summarizing the embodiment/presence constructs and two components summarizing the behavioral measures. The PLS-SEM was estimated on the reduced dataset.

All statistical analyses have been performed using R studio version 2023.09.1 (https://rstudio.com/)

#### 2.6.1. Analysis of variance (ANOVA)

We performed a repeated measures analysis of variance with the *session* (active vs sham) as the within-subject factor and the *order* of sessions (active first, sham first) as the between-subject factor. Introducing the factor *order* allows for control over any potential learning effects between sessions, despite our efforts to pseudo-randomize and balance the order of sessions over the subjects.

#### 2.6.2. Exploratory and confirmatory factor analysis (EFA and CFA)

We performed the EFA on behavioral indices to identify a reliable factorial structure of performance measures (driving inaccuracy, elapsed time, duration of time lapses, short-term self-similarity; long-term self-similarity). Similarly, we performed the EFA on the factors of Presence and Avatar Embodiment questionnaires to identify a higher-order construct capable of synthesizing the key features of these questionnaires while mitigating multicollinearity between questionnaires.

For EFA, the varimax rotation method was used to better contrast loadings between items belonging to each factor. Items were retained as belonging to a factor when the absolute value of the corresponding loading was greater than 0.30.

After conducting EFA, we proceeded with the CFA to validate the structural integrity of the factorial models [45]. For the model derived from the Presence and Embodiment questionnaires, a second-order CFA was used, as EFA had been applied to the validated factors of the Avatar Embodiment and Presence Questionnaire, as mentioned in sections 2.5.1 and 2.5.2 questionnaire [46],[47].

From EFA, regarding behavioral indices of performance, we identified two factors: inaccuracy (INACC1 = driving inaccuracy, INACC2 = elapsed time) and stability (STAB1 = short-term self-similarity; STAB2 = long-term self-similarity, STAB3 = duration of time lapses) factor. CFA indicated a good factor model fitting (Comparative Fit Index = 1.00; Tucker-Lewis Index = 1.152; Standardized Root Mean Square Residual = 0.042). Concerning Avatar Embodiment and the Presence questionnaires, we identified two factors: Embodiment (EMB1 = ownership, EMB2 = agency, EMB3 = location) and presence (PRES1 = quality of interface, PRES2 = possibility to act, PRES3 = possibility to examine). Second-order CFA indicated a good factor model fitting (Comparative Fit Index = 1.00; Tucker-Lewis Index = 1.012; Standardized Root Mean Square Residual = 0.059).

Regarding the NASA TLX, we maintained the number of factors identified by the original version [42] (DEM1 = mental demand, DEM2 = physical demand, DEM3 = temporal demand, DEM4 = own performance, DEM5 = effort, DEM6 = frustration).

#### 2.6.3. Partial Least Squares Structural Equation Modeling

**Figure 4.**
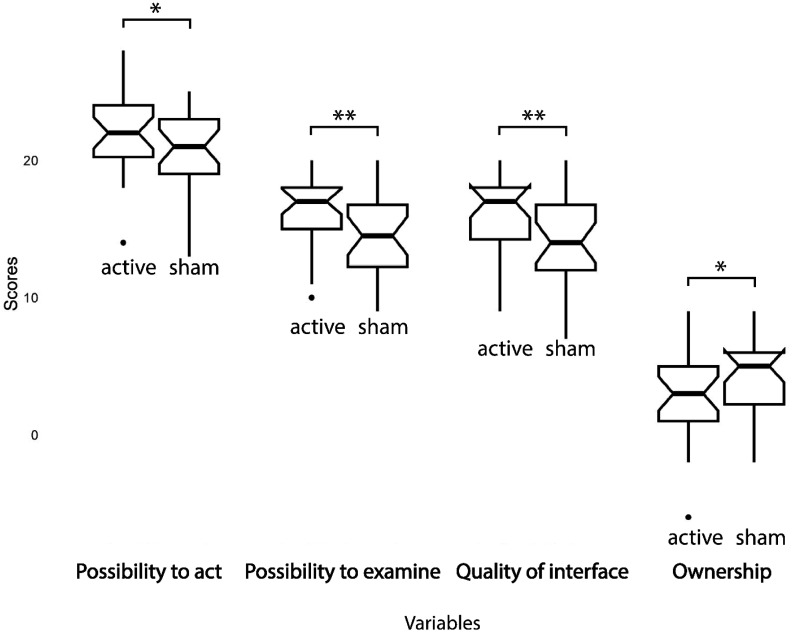
Boxplots of significant mean differences of psychometric questionnaires between active rTPJ stimulation and sham sessions (*= p ≤0.05; **= p ≤ 0.01; *** = p ≤ 0.001).

We estimated causal and simultaneous relationships among psychometric and behavioral indices of performance *via* Partial Least Squares Structural Equation Modeling (PLS-SEM). A structural equation model comprises measurement models and structural models that are simultaneously estimated and interconnected. A measurement model shows the relationship between each observed variable and its latent variable (represented by the relationships between rectangle and hexagon blocks in Figure 4). A structural model illustrates the relationships between latent variables (depicted by the relationships between hexagon blocks in Figure 4).

To estimate the coefficients linking observable to latent variables (λ) and different latent variables (β), Partial Least Squares SEM was employed since it addresses non-normal datasets and small sample size and is recommended for exploratory research [48].

With PLS-SEM, the reliability of the model is evaluated using composite reliability (RhoC) and Cronbach's alpha, which should exceed 0.7 [49].

Besides, a new reliability coefficient (RhoA) was computed as it would better reflects the actual reliability of construct scores compared to Cronbach's alpha, which tends to underestimate, and RhoC, which tends to overestimate the actual reliability [50]. In addition, the average variance extracted (AVE) was calculated for convergent validity estimates, with an expected value greater than 0.5 [49].

PLS-SEM analysis was computed with the SEMinR package [49] in R studio.

As our study consisted of two paired sessions, we estimated the PLS-SEM on both sham and active sessions, and then we used a modified multigroup analysis for testing differences between sessions, that is to identify an effect of the rTPJ stimulation on the structure model. This approach was introduced by Söllner and colleagues [51] who used a novel multi-group analysis (MGA) based on an adaptation of the permutation approach to repeated measures. This approach has allowed us to assess the changes in the strength of different relationships over different conditions, studying a group of individuals multiple times.

In the model implemented in this work, the embodiment was set as the exogenous variable, functioning as the independent variable expected, according to the hypothesis to be verified, to influence the other variables (endogenous variables) without being influenced by them. In contrast, endogenous variables (sense of presence, workload, inaccuracy, and stability) were assumed to be influenced by other variables in the model, including both endogenous and exogenous variables.

It is noteworthy that these causal directions have been obtained according to previous studies [6],[8],[9],[52]–[54], which argue for the causal role of the embodiment and the sense of presence on both subjective (workload) and objective behavioral indices of performance.

Based on the lack of differences between sessions derived by applying MGA, the final SEM was estimated on the pooled dataset.

## 3. Results

### 3.1. rTPJ Stimulation: Major effects

The repeated measures ANOVA yielded the following statistically significant results. Regarding behavioral outcomes, short-term self-similarity and long-term self-similarity values were higher in active sessions (df (1,24); F = 3.74; p = 0.05; ηp^2^ = 0.18; df (1,24); F = 5.62; p = 0.026; ηp^2^ = 0.19, respectively). No significant changes were noted in the following performance indices related to attentional functioning: driving inaccuracy; elapsed time in the lap; and duration of attentional lapses.

Concerning the Avatar Embodiment questionnaire, the *sense of ownership* decreased in the active session (df (1,24); F =7.19; p = 0.013; ηp^2^ = 0.23). As for the Presence questionnaire, the *possibility to act* was higher in the active session (df (1,24), F = 3.59; p = 0.04; ηp^2^ = 0.17), along with the *possibility to examine* (df (1,24); F =8.92; p = 0.006; ηp^2^ = 0.27) and the *quality of interface* (df (1,24); F =8.33; p = 0.008; ηp^2^ = 0.25).

**Figure 5.**
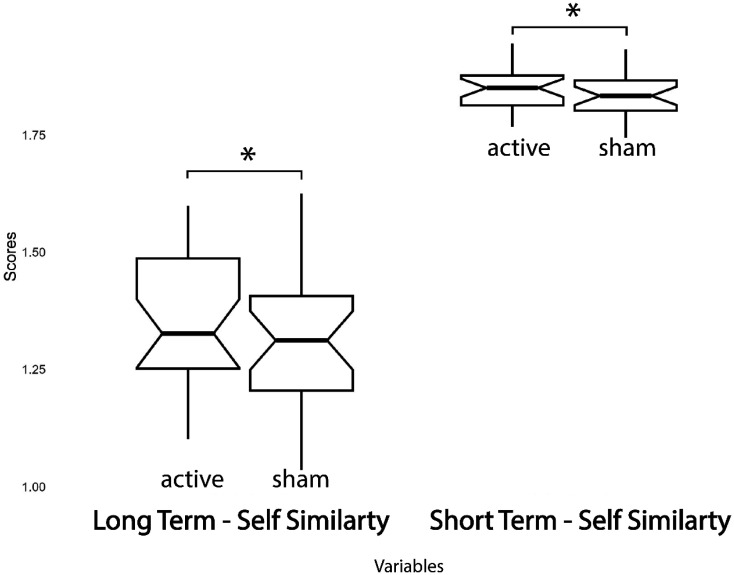
Boxplots of significant differences in behavioral measures between active and sham sessions (*= p ≤0.05; **= p ≤ 0.01; *** = p ≤ 0.001).

Neither the order of session factor nor the interaction between order and session factors was significant for any variable. Refer to Figure 4 and Figure 5 for a graphical representation of significant differences.

#### 3.1.1. Sensations Experienced During tDCS Stimulation

Participants reported varying sensations during the tDCS stimulation, assessed using a brief follow-up interview based on an Adverse Effects Questionnaire adapted from Poreisz et al. [44]. The reported sensations that could be perceived during both the active and sham stimulation sessions (mind tingling, itchiness under the electrode sites) were generally brief and did not interfere with task performance or participants' engagement in the study.

### 3.2. Interplay between embodiment, presence, and performance: Structural Equation Modeling

**Figure 6.**
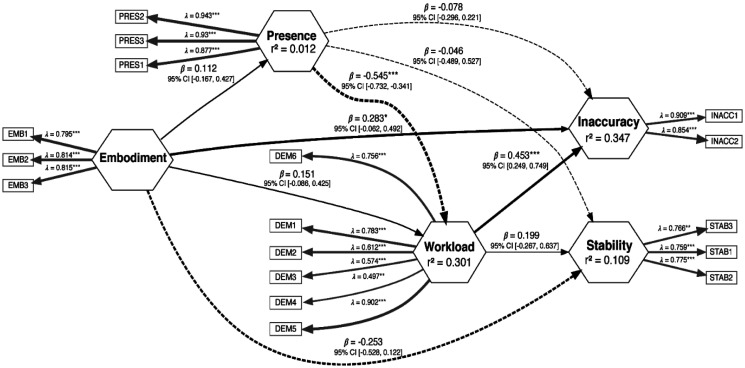
Partial Least Squares Structural Equation Modeling among exogenous and endogenous variables (*= p ≤0.05; **= p ≤ 0.01; *** = p ≤ 0.001). Notes: *EMB*: embodiment; *PRES*: presence; *DEM*: demand; *INACC*: inaccuracy; *STAB*: stability.

The PLS model was estimated on the entire dataset according to the results of multigroup analysis (MGA). Figure 6 shows the emergent structure, revealing that the level of Presence negatively influenced Workload (β = -0.545; p < 0.001; *f^2^* = 0.44), while Embodiment (β = 0.283; p < 0.05; *f^2^* = 0.16) and the Workload (β = 0.453; p < 0.001; *f^2^* = 0.34) positively affected Inaccuracy.

Construct reliability for the model was assessed by examining composite reliability (RhoC), actual variability (RhoA), Cronbach Alpha, and average variance extracted (AVE). All the reliability indices satisfied the criteria for acceptability (Table 1), indicating the reliability of the model.

**Table 1.** Reliability Indices of PLS Structural Equation Model.

	Alpha	RhoC	AVE	RhoA
Embodiment	0.73	0.85	0.65	0.74
Presence	0.98	0.94	0.84	0.94
Workload	0.72	0.84	0.50	0.85
Accuracy	0.71	0.87	0.77	0.74
Stability	0.70	0.81	0.58	0.65

## 4. Discussion

This study aimed to investigate the impact of tDCS on various aspects of human performance, including embodiment, presence, cognitive workload, and objective task performance. To achieve this goal, participants underwent a VR driving task twice, with targeted tDCS applied to the rTPJ during one session.

### 4.1. Feeling there, being here: Temporo-parietal-junction modulation might favor the sense of telepresence through (dis)embodiment

When comparing active and sham sessions, we observed that participants reported a heightened sense of presence during active stimulation. Specifically, they expressed greater engagement with the immersive scenario, suggesting the role of TPJ stimulation in fostering the sensation of “Feeling there, being here”, a concept previously described as telepresence by Minsky [55].

Conversely, participants reported a decreased level of experienced ownership during active sessions, indicating a reduced sense of incorporating non-bodily objects, such as tools, into one's body. These findings partially align with previous research emphasizing the primary role of the TPJ in promoting altered states of consciousness [12]. The sense of presence can be described as an altered state of consciousness, which intriguingly resembles an artificial form of “out-of-the-body experience” [11],[56], and accordingly, it is modified by the TPJ stimulation, a key structure located in the posterior cortical “hot zone” of the brain [57],[58], a critical area within the posterior cerebral cortex that is thought to be a key region for the neural correlates of consciousness and capable of influencing the sense of (tele)presence[57],[59]. Research has shown that applying non-invasive brain stimulation to the TPJ can induce feelings of disembodiment by altering body boundaries, thereby leading to several changes in self-perception or perception of others' bodies. For instance, studies have demonstrated that noninvasive brain modulation techniques applied to TPJ can impair the mental transformation of one's own body [12], proprioceptive abilities [60], and the resolution of intersensory conflicts [61].

When stimulating this region, participants may have experienced “body transportation” to become immersed in a remote virtual environment (enhancing active presence), potentially at the cost of perceiving virtual tools as part of their own body (reducing ownership).

This dissociation observed between telepresence and ownership following TPJ stimulation may be partially explained by the notion that a heightened sense of ownership in a VR driving task, which demands visuomotor performance, could impede optimal task performance. In other words, ownership is believed to play a more significant role in integrating a virtual body rather than in actual action performance. This discrepancy could stem from the differentiation between two types of body representations: a body schema related to visuomotor actions (as observed in our driving task), and a body image associated with proprioception, primarily influenced by the visual representation of the virtual avatar [62].

From a behavioral perspective, the only parameters influenced by rTPJ stimulation are the short and long-term self-similarity of the departures time series. According to previous literature, this result suggested a heightened level of integration during predictive motor coding due to the stimulation of the rTPJ hub [63]. Surprisingly, our study did not yield significant major effects of tDCS on attentional indices and visuomotor control measures (driving inaccuracy, elapsed time in the lap, time spent in attentional lapses). Additionally, no change in perceived workload was observed in association with the stimulation. This suggests that, in our study, the tDCS application did not significantly impact the specific attentional processes or perceived cognitive load as reported by Wu et al. [64], who demonstrated the role of tDCS in modulating attentional control by influencing cognitive load and stimulus surprise, as well as functional connectivity between the TPJ and other brain regions involved in attentional processes. It is also worth to note that the lack of a significant effect of tDCS on visuomotor control may be influenced by the placement of the return electrode (cathode) over CZ, which could impair visuomotor coordination, and this effect is likely due to reduced motor cortex excitability induced by cathodal tDCS [65].

### 4.2. Striking a balance in virtual reality: How presence eases workload but embodiment challenges accuracy

The results of the structural equation model indicate a causal relationship between telepresence and perceived workload, and between workload and performance. Specifically, the sense of presence reduced the perceived workload, and the higher perceived workload increased performance inaccuracy. Moreover, embodiment was found to increase performance inaccuracy.

Regarding the relationship between embodiment and inaccuracy, our results partially contradict previous literature that indicated that the sense of embodiment enhances subjective performance [6]. For instance, some studies reported that the degree of embodiment over a virtual limb directly modulates performance in simple sensorimotor tasks with higher levels of embodiment leading to faster reaction times [66]–[68].

It is noteworthy that the role of embodiment in performance has been relatively understudied, primarily investigated in the context of body illusions, such as Rubber Hand Illusion, without considering performance indices. Therefore, while the sense of embodiment may be crucial for experiencing oneself as an avatar, it could also be a detrimental factor for performance. Perceived embodiment towards the avatar (in our study the virtual hand that controls the vehicle) may divert attention towards the virtual effector, potentially impairing visuomotor performance. An alternative explanation could be related to the multisensory environment provided by VR. Despite the multiple potentials of using tactile feedback to enhance the sense of embodiment [6],[69],[70], the artificial nature of this feedback delivery method could influence the perception of control and negatively impact performance [71].

It could be argued that somatosensory feedback may not reach the adequate level of fidelity required to replicate interactions in the natural environment [72]. Furthermore, the issue of dimensionality [72] should be considered, referring to the accuracy with which interaction methods in VR replicate the control dimensions of the real-world task. This dimensionality may not be as present in virtual environments compared to physical ones. For instance, in fields like robotic surgery, where telemanipulation is crucial, the introduction of haptic feedback has been found to impair surgical performance [73].

In summary, a negative causal relationship between embodiment and accuracy may be due to two major features: first, the allocation of individual attentional resources towards the virtual avatar, which leads to the prioritization of avatar perception and integration and relegates task completion to a secondary role; second, the artificial nature of delivered feedback during task completion, which may yield dissociable outcomes: enhancing the sense of embodiment on the one hand and exerting a detrimental effect on visuomotor performance on the other hand.

Concerning the negative relationship between telepresence and workload, previous studies have shown contradictory results: some of them found that the degree of presence experienced in gaming and cinema sectors is positively associated with workload [52],[54],[74], and it has been proposed that, in the field of virtual and immersive scenarios, the simultaneous use of multiple sensory channels could facilitate an increase of the sense of presence at the expense of a higher perceived workload.

Evidence suggests a positive impact of presence on workload, with higher presence reducing frustration during tasks [75],[76]. However, the complex relationship between presence and workload, particularly in telerobotics, is partially understood.

Our results revealed a negative causal relationship between presence and workload, potentially explained by the sense of (tele)presence promoting an effortless state of consciousness. In fact, (tele)presence can be viewed as akin to an out-of-body experience [11],[56], where body boundaries are altered to create a sense of “Feeling there, being here”. This state enables individuals to focus on reshaping body boundaries, fostering a sense of presence in the virtual environment despite physical distance, and diverting attention from self-reflective thoughts on task performance.

Importantly, rTPJ stimulation did not alter these relationships, indicating structural invariance across sessions. While our study is novel in incorporating tDCS for multigroup analysis, previous research has also highlighted similar relationships between presence, workload, and performance, partially supporting our findings [6],[52]–[54].

In summary, the sense of presence may have a positive impact on workload by inducing an altered state of consciousness that directs individuals' attention away from performance concerns and towards the immersive experience of being transported into another environment (the virtual one). However, this heightened sense of presence does not necessarily lead to performance optimization, as the primary focus remains on the process itself rather than on task performance.

## 5. Conclusions

We highlight, for the first time, the effect of tDCS in decreasing body ownership and enhancing the sense of (tele)presence and visuomotor control. Additionally, the role of embodiment in decreasing performance represents an innovative perspective, wherein the subjective effort to integrate avatar perception may take precedence over human performance. The subjective effort to integrate these tools can serve as a distraction from achieving proficient performance. On the other hand, the reduced workload resulting from the impact of the sense of presence could potentially counteract the subjective perception of high demand during task completion, thus facilitating engagement with the task.

## References

[1] Cui J, Tosunoglu S, Roberts R (2003). A review of teleoperation system control. In: Proceedings of the Florida conference on recent advances in robotics; Boca Raton, FL.

[2] Pala M, Lorencik D, Sincak P (2012). Towards the robotic teleoperation systems in education. In: 2012 IEEE 10th International Conference on Emerging eLearning Technologies and Applications (ICETA); Stará Lesná, Slovakia.

[3] Siciliano B, Khatib O (2016). Springer Handbook of Robotics.

[4] Schinstock DE (1998). Approximate solutions to unreachable commands in teleoperation of a robot. Robot Comput Integr Manuf.

[5] Álvarez B, Iborra A, Alonso A (2001). Reference architecture for robot teleoperation: development details and practical use. Control Eng Pract.

[6] Toet A, Kuling IA, Krom BN (2020). Toward Enhanced Teleoperation Through Embodiment. Front Robot AI.

[7] Kilteni K, Groten R, Slater M (2012). The Sense of Embodiment in Virtual Reality. Presence Teleoperators Virtual Environ.

[8] Slater M (2009). Place illusion and plausibility can lead to realistic behaviour in immersive virtual environments. Philos Trans R Soc Lond B Biol Sci.

[9] Slater M, Spanlang B, Corominas D (2010). Simulating virtual environments within virtual environments as the basis for a psychophysics of presence. ACM Trans Graph.

[10] Lombard M, Biocca F, Freeman J (2015). Immersed in Media: Telepresence Theory, Measurement & Technology.

[11] Herbelin B, Salomon R, Serino A (2016). 5. Neural Mechanisms of Bodily Self-Consciousness and the Experience of Presence in Virtual Reality. Human Computer Confluence: Transforming Human Experience Through Symbiotic Technologies.

[12] Blanke O, Mohr C, Michel CM (2005). Linking out-of-body experience and self processing to mental own-body imagery at the temporoparietal junction. J Neurosci Off J Soc Neurosci.

[13] Orrù G, Bertelloni D, Cesari V (2021). Targeting temporal parietal junction for assessing and treating disembodiment phenomena: a systematic review of TMS effect on depersonalization and derealization disorders (DPD) and body illusions. AIMS Neurosci.

[14] Arzy S, Thut G, Mohr C (2006). Neural basis of embodiment: distinct contributions of temporoparietal junction and extrastriate body area. J Neurosci Off J Soc Neurosci.

[15] Ptak R, Schnider A (2011). The attention network of the human brain: Relating structural damage associated with spatial neglect to functional imaging correlates of spatial attention. Neuropsychologia.

[16] Donaldson PH, Rinehart NJ, Enticott PG (2015). Noninvasive stimulation of the temporoparietal junction: A systematic review. Neurosci Biobehav Rev.

[17] Pascual-Leone A, Amedi A, Fregni F (2005). The plastic human brain cortex. Annu Rev Neurosci.

[18] Polanía R, Nitsche MA, Ruff CC (2018). Studying and modifying brain function with non-invasive brain stimulation. Nat Neurosci.

[19] Santarnecchi E, Brem A-K, Levenbaum E (2015). Enhancing cognition using transcranial electrical stimulation. Curr Opin Behav Sci.

[20] Škola F, Liarokapis F (2021). Study of Full-body Virtual Embodiment Using noninvasive Brain Stimulation and Imaging. Int J Hum-Comput Interact.

[21] Convento S, Romano D, Maravita A (2018). Roles of the right temporo-parietal and premotor cortices in self-location and body ownership. Eur J Neurosci.

[22] Lapenta OM, Fregni F, Oberman LM (2012). Bilateral temporal cortex transcranial direct current stimulation worsens male performance in a multisensory integration task. Neurosci Lett.

[23] Marques LM, Lapenta OM, Merabet LB (2014). Tuning and disrupting the brain—modulating the McGurk illusion with electrical stimulation. Front Hum Neurosci.

[24] Lira M, Pantaleão FN, de Souza Ramos CG (2018). Anodal transcranial direct current stimulation over the posterior parietal cortex reduces the onset time to the rubber hand illusion and increases the body ownership. Exp Brain Res.

[25] Abedanzadeh R, Alboghebish S, Barati P (2021). The effect of transcranial direct current stimulation of dorsolateral prefrontal cortex on performing a sequential dual task: a randomized experimental study. Psicol Reflexao E Crit Rev Semest Dep Psicol UFRGS.

[26] Fregni F, Liguori P, Fecteau S (2008). Cortical stimulation of the prefrontal cortex with transcranial direct current stimulation reduces cue-provoked smoking craving: a randomized, sham-controlled study. J Clin Psychiatry.

[27] Mondino M, Luck D, Grot S (2018). Effects of repeated transcranial direct current stimulation on smoking, craving and brain reactivity to smoking cues. Sci Rep.

[28] Stagg CJ, Jayaram G, Pastor D (2011). Polarity and timing-dependent effects of transcranial direct current stimulation in explicit motor learning. Neuropsychologia.

[29] Nitsche MA, Paulus W (2000). Excitability changes induced in the human motor cortex by weak transcranial direct current stimulation. J Physiol.

[30] Khalil R, Karim AA, Godde B (2023). Less might be more: 1 mA but not 1.5 mA of tDCS improves tactile orientation discrimination. IBRO Neurosci Rep.

[31] Santiesteban I, Banissy MJ, Catmur C (2012). Enhancing Social Ability by Stimulating Right Temporoparietal Junction. Curr Biol.

[32] Santiesteban I, White S, Cook J (2012). Training social cognition: From imitation to Theory of Mind. Cognition.

[33] Bufano P, Albertini N, Chiarelli S (2022). Weakened Sustained Attention and Increased Cognitive Effort after Total Sleep Deprivation: A Virtual Reality Ecological Study. Int J Human–Computer Interact.

[34] Cesari V, Marinari E, Laurino M (2021). Attention-Dependent Physiological Correlates in Sleep-Deprived Young Healthy Humans. Behav Sci Basel Switz.

[35] Laurino M, Menicucci D, Mastorci F (2012). Mind-body relationships in elite apnea divers during breath holding: a study of autonomic responses to acute hypoxemia. Front Neuroengineering.

[36] Peng CK, Havlin S, Stanley HE (1995). Quantification of scaling exponents and crossover phenomena in nonstationary heartbeat time series. Chaos Woodbury N.

[37] Maczák B, Gingl Z, Vadai G (2024). General spectral characteristics of human activity and its inherent scale-free fluctuations. Sci Rep.

[38] Gonzalez-Franco M, Peck TC (2018). Avatar Embodiment. Towards a Standardized Questionnaire. Front Robot AI.

[39] Klotz AC, Swider BW, Kwon SH (2023). Back-translation practices in organizational research: Avoiding loss in translation. J Appl Psychol.

[40] Cyberpsychology Lab of UQO, ‘Welcome to the Cyberpsychology Lab of UQO!’, consulted on 9 May 2024. [Online].

[41] Witmer BG, Singer MJ (1998). Measuring presence in virtual environments: A presence questionnaire. Presence Teleoperators Virtual Environ.

[42] Hart SG, Staveland LE (1988). Development of NASA-TLX (Task Load Index): Results of empirical and theoretical research. Human mental workload.

[43] Mueller ST, Piper BJ (2014). The Psychology Experiment Building Language (PEBL) and PEBL Test Battery. J Neurosci Methods.

[44] Poreisz C, Boros K, Antal A (2007). Safety aspects of transcranial direct current stimulation concerning healthy subjects and patients. Brain Res Bull.

[45] Harrington D (2009). Confirmatory Factor Analysis.

[46] Brown TA (2015). Confirmatory Factor Analysis for Applied Research, Second Edition, Guilford Publications.

[47] Gould SJ, Hawes JM, Glisan GB (2015). Second Order Confirmatory Factor Analysis: An Example. Proceedings of the 1987 Academy of Marketing Science (AMS) Annual Conference.

[48] Jöreskog KG, Wold HOA (1982). Systems Under Indirect Observation: Causality, Structure, Prediction, North-Holland.

[49] Hair JF, Hult GTM, Ringle CM, Hair JF, Hult GTM, Ringle CM (2021). The SEMinR Package. Partial Least Squares Structural Equation Modeling (PLS-SEM) Using R: A Workbook.

[50] Dijkstra TK, Henseler J (2015). Consistent Partial Least Squares Path Modeling. MIS Q.

[51] Söllner M, Mishra AN, Becker J-M (2024). Use IT again? Dynamic roles of habit, intention and their interaction on continued system use by individuals in utilitarian, volitional contexts. Eur J Inf Syst.

[52] Breves P, Schramm H (2019). Good for the feelings, bad for the memory: the impact of 3D versus 2D movies on persuasion knowledge and brand placement effectiveness. Int J Advert.

[53] Buonocore S, Massa F, Di Gironimo G, Ahram Tareq, Karwowski Waldemar (2023). Does the Embodiment Influence the Success of Visuo-haptic Learning?. Application of Emerging Technologies. AHFE (2023) International Conference. AHFE Open Access, vol 115.

[54] Roettl J, Terlutter R (2018). The same video game in 2D, 3D or virtual reality – How does technology impact game evaluation and brand placements?. PLOS ONE.

[55] Minsky M (1980). Telepresence. OMNI Magazine.

[56] Rheingold H (1991). Virtual Reality: Exploring the Brave New Technologies, Simon & Schuster Adult Publishing Group.

[57] Koch C, Massimini M, Boly M (2016). Neural correlates of consciousness: progress and problems. Nat Rev Neurosci.

[58] Tononi G, Boly M, Gosseries O (2016). The Neurology of Consciousness.

[59] Dai R, Larkin TE, Huang Z (2023). Classical and non-classical psychedelic drugs induce common network changes in human cortex. NeuroImage.

[60] Tsakiris M, Costantini M, Haggard P (2008). The role of the right temporo-parietal junction in maintaining a coherent sense of one's body. Neuropsychologia.

[61] Papeo L, Longo MR, Feurra M (2010). The role of the right temporoparietal junction in intersensory conflict: detection or resolution?. Exp Brain Res.

[62] Kammers MPM, Longo MR, Tsakiris M (2009). Specificity and Coherence of Body Representations. Perception.

[63] Jakobs O, Wang LE, Dafotakis M (2009). Effects of timing and movement uncertainty implicate the temporo-parietal junction in the prediction of forthcoming motor actions. NeuroImage.

[64] Wu Q, Chang C-F, Xi S (2015). A critical role of temporoparietal junction in the integration of top-down and bottom-up attentional control. Hum Brain Mapp.

[65] Nitsche MA, Nitsche MS, Klein CC (2003). Level of action of cathodal DC polarisation induced inhibition of the human motor cortex. Clin Neurophysiol Off J Int Fed Clin Neurophysiol.

[66] Berg H (2019). How does evidence-based practice in psychology work? – As an ethical demarcation. Philos Psychol.

[67] Grechuta K, Guga J, Maffei G (2017). Visuotactile integration modulates motor performance in a perceptual decision-making task. Sci Rep.

[68] Kokkinara E, Slater M, Lopez-Moliner J (2015). The Effects of Visuomotor Calibration to the Perceived Space and Body, through Embodiment in Immersive Virtual Reality. ACM Trans Appl Percept.

[69] Tsakiris M, Haggard P (2005). The Rubber Hand Illusion Revisited: Visuotactile Integration and Self-Attribution. J Exp Psychol Hum Percept Perform.

[70] Botvinick M, Cohen J (1998). Rubber hands ‘feel’ touch that eyes see. Nature.

[71] Cesari V, D'Aversa S, Piarulli A (2024). Sense of Agency and Skills Learning in Virtual-Mediated Environment: A Systematic Review. Brain Sci.

[72] Levac DE, Huber ME, Sternad D (2019). Learning and transfer of complex motor skills in virtual reality: a perspective review. J NeuroEngineering Rehabil.

[73] El Rassi I, El Rassi J-M (2020). A review of haptic feedback in tele-operated robotic surgery. J Med Eng Technol.

[74] Ma R, Kaber DB (2006). Presence, workload and performance effects of synthetic environment design factors. Int J Hum-Comput Stud.

[75] Kaber DB, Riley JM (1999). Adaptive automation of a dynamic control task based on secondary task workload measurement. Int J Cogn Ergon.

[76] Lackey SJ, Salcedo JN, Szalma JL (2016). The stress and workload of virtual reality training: the effects of presence, immersion and flow. Ergonomics.

